# Natural Breeding Places for Phlebotomine Sand Flies (Diptera: Psychodidae) in a Semiarid Region of Bahia State, Brazil

**DOI:** 10.1155/2012/124068

**Published:** 2012-02-28

**Authors:** Bruno Sangiorgi, Daniel Neves Miranda, Diego Ferreira Oliveira, Edivaldo Passos Santos, Fernanda Regis Gomes, Edna Oliveira Santos, Aldina Barral, José Carlos Miranda

**Affiliations:** ^1^Centro de Pesquisas Gonçalo Moniz/FIOCRUZ, 40295-001, Salvador, BA, Brazil; ^2^Faculdade de Medicina da Bahia, Universidade Federal da Bahia, 40025-010, Salvador, BA, Brazil; ^3^Instituto de Investigação em Imunologia, Instituto Nacional de Ciência e Tecnologia, Ciencia e Tecnologia (INCT), 05403-900 Cerqueira Cesar, SP, Brazil

## Abstract

Few microhabitats have been previously identified as natural breeding places for phlebotomine sand flies so far, and little is known about the influence of climate variables in their density. The present study was conducted in a dry region with a semiarid climate, where visceral leishmaniasis occurs in humans and dogs. The occurrence of breeding places in specific microhabitats was investigated in soil samples collected from five houses, which were also the location used for sampling of adults. All the microhabitats sampled by our study were identified as natural breeding places due to the occurrence of immature forms of sand flies. On a weekly basis, the number of adult sand flies captured was positively correlated with the mean temperature from preceding weeks. These results, in addition to promoting an advance in the knowledge of sand flies biology, may furnish a tool for optimizing the control of the sand flies, by indicating the most suitable periods and microhabitats for the application of insecticides.

## 1. Introduction

Despite the medical importance of leishmaniasis, little is known about the natural breeding places of its vectors. Most previous attempts to identify the preferred microhabitats for the oviposition of sand flies in the Neotropical region have produced disappointing yields, resulting in a small number of positive soil samples and immature forms [1–4]. Some recent studies (e.g., Alencar [5] and Singh [6]), however, have successfully obtained high amounts of immature forms due to the sampling of suitable places for larval development.

As observed by Newstead [7], the immature forms of sand flies are more concentrated in microhabitats that exhibited specific conditions, as the presence of organic matter, humidity, and low levels of light. Studies conducted in rain forests (e.g., Hanson [8], Alencar et al. [5]) corroborate these observations, as shown by the greater numbers of immature forms found in soil with litter, between roots and under fallen trunks. Similar edaphic conditions, although in different microhabitats such as soil cracks, have also been observed in studies conducted at regions with dry climates (e.g., Deane and M. P. Deane [9], Ferreira et al. [1]), to be more likely to find immature forms of sand flies.

Dry climate regions experience more pronounced climatic variations than regions with humid climates typical of rain forests, so the density of vectors may exhibit different characteristics as well. Studies conducted in rain forest regions (e.g., Hanson [8], Dias-Lima et al. [10]) observed high densities of vectors throughout the entire study period. In contrast, studies from dry climate regions presented controversial results, as the absence of correlation between climate variables and sand flies density [11, 12], or increasing of density soon after rainy periods [13, 14].

The knowledge about natural breeding places for sand flies and vector density may represent useful information for directing efforts at biological control, leading to reduce the density of the vectors and consequently control the incidence of disease [15, 16]. In the present study, we aimed to characterize the natural breeding places for sand flies in a region with a dry climate and a known incidence of visceral leishmaniasis during the past 20 years. Additionally, we investigated the possibility of association between the density of adults or immature sand flies with climate variables, assessing the predictive value of these variables on sand flies density.

## 2. Methods

### 2.1. Area of Study

**Figure 1:**
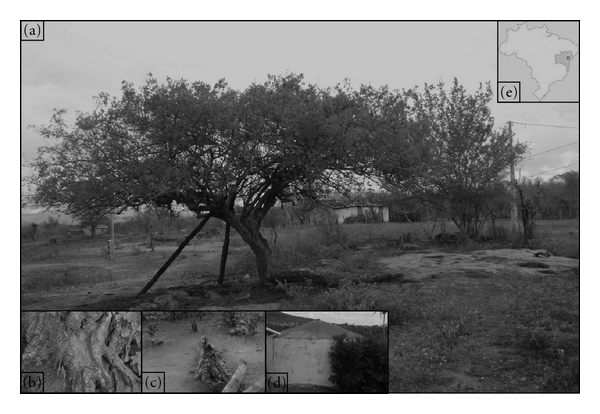
Location of Cavunge district and positive microhabitats (a) Typical scenario from Cavunge: trees with exposed roots along with exposed rocks (b) soil accumulated between exposed roots of a *Spondias tuberosa* tree (c) a fallen trunk (d) a water tank. (e) location of Cavunge district.

The Cavunge district (12.3°S; 39.3°W) is located in a semiarid region of Bahia state (Figure 1). This district has 63.5 km² of area in which the Caatinga, a type of vegetation characterized by dispersed bushes and deciduous trees, is the major biome. Breeding sites were searched in five residences, as the owners allowed samplings both inside and in places adjacent to their houses.

### 2.2. Soil Sampling

During the first period of this study, from June 2007 through July 2008, soil samples at low-light places with apparent decomposed organic matter, were collected from areas immediately adjacent to the five residences selected by our study group. These samples were gathered with a regular spoon and stored in cylindrical receptacles with a capacity of 1 L. Microhabitats from which positive samples were collected were then chosen for more intensive sampling during the subsequent study period.

During the second period of our study, from August 2008 through July 2010, approximately 64 soil samples were monthly collected at the same five residences and stored in similar receptacles. According to the results from the first year of study, the soil was sampled in the following microhabitats: cavities in rocks; areas between the roots of a *Spondias tuberosa* tree; areas between the roots of a *Delonix regia *tree; cavities in a fallen tree trunk; cracks around a water tank; areas covered with chicken feces; low-light places inside a house (e.g: hollows in walls, floor and around clay jugs). All soil samples were sent to an insectarium at the Centro de Pesquisas Gonçalo Moniz (CPqGM), being analyzed with different techniques.

### 2.3. Processing of Soil Samples

 During the entire period of study, three different techniques: direct-observation, flotation-sieving, and flotation technique were utilized to identify the presence of sand flies in the soil samples.

 The direct-observation technique was used to examine all soil samples until sixty days after their arrival at the insectarium. This technique consisted by daily observation of the receptacles containing the soil samples in two different ways: by naked eye, so to find adult sand flies through the translucent cover of the 1L receptacles, and with a stereomicroscope, aiming to find immature forms in the soil. To count the number of sand flies in a soil sample, we included immature forms that reached the adult stage, immature forms that did not reach the adult stage but were similar to other larval stages (larvae and pupae) present in our laboratory colony of *L. longipalpis*, and the adults whose previous larval stages had not been detected.

After the period corresponding to the direct observation technique, all soil samples were submitted to the flotation-sieving technique or the flotation technique.

The flotation-sieving technique, as described by Hanson [8], was used only in the soil samples from the first part of the study. According to this technique, each soil sample was removed from the receptacles and disposed in a sieve. The soil was then washed with tap water through the sieve and through another two sieves, consecutively, with smaller pores each. The material that passed through the three sieves was then suspended with a saturated sugar solution, in order that immature forms could be found floating in the high-density solution.

 Due to the retention of immature forms on the sieves used to process the soil samples from the first part of the study, and consequently loss of information about the real quantity of immature forms in the soil samples, the sieves were replaced by flotation for the second part of the study. With the flotation technique, a saturated salt solution was simply added to the soil samples and allowed the immature forms to float.

Following floatation-sieving and flotation, the immature forms suspended by a high density solution, were collected with a brush and disposed separately in 1L capacity cylindrical receptacles, remaining there for 60 days. These vessels were one-third filled with plaster covered by a ration composed by a mixture of rabbit feces, commercial hamster ration and small quantity of soil from the original substrate. The 2 inch hole in the bottom of these containers allowed keeping the plaster constantly humid due to the directly contact with a moist paper towel.

The adult sand flies gathered from direct observation, flotation-sieving and flotation techniques were stored in covered glass slides with mounting media and identified at species level based on the identification key proposed by Young and Duncan [17].

### 2.4. Sampling of Adults

 During each month of the second part of the study, one day before the soil sampling, two HP light traps, one inside the residence, and another in the adjacencies, were placed in every one of the same five residences used for soil sampling. The light traps were operated from 6 pm to 6 am, period also used by other authors due to the known nocturnal activity of several sand flies species [18, 19]. All captured sand flies were transferred to the sand fly insectarium at CPqGM. At the arrival moment, adults were individually stored in covered glass slides with mounting media, following by identification at species level based on the identification key proposed by Young and Duncan [17].

### 2.5. Climate Data

 Climate data were obtained by the National Institute of Meteorology (INMET) from the Feira de Santana meteorological station, placed at about 40 km of distance from Cavunge district. Daily means from temperature, and relative humidity were provided directly from the INMET, which calculated these data through the arithmetic means from records of maximum and minimum temperature or relative humidity at 9 am, 3 pm and 9 pm, respectively. Otherwise, daily rainfall was measured by a single record of millimeters of rain in a day.

Daily rainfall and daily averages for temperature, and relative humidity were used to obtain the averages of these variables on a weekly basis. Weekly averages of rainfall, temperature and relative humidity were calculated through the arithmetic means of daily rainfall, mean temperature and mean relative humidity, respectively, at one up to three weeks before the soil or adult sampling days. Thus, the density of immature or adult sand flies was correlated by statistical analyses with the preceding weekly averages from the climate variables.

### 2.6. Statistical Analyses

From Minitab software, multiple linear regression analyzes were performed for the yield of sand flies in the soil or adult sampling in each month and the average of climate variables at one up to three weeks before soil or adult sampling days.

## 3. Results

During the first period of this study, 234 soil samples were collected from five residences in the Cavunge district. Nine of these samples were identified as positives by direct-observation or flotation-sieving technique in the microhabitats and included rock cavities (2), between the roots of *Spondias tuberosa (2)*, between the roots of *Delonix regia *(1), soil cracks around a water tank (2), soil covered with chicken feces (2). Based on these results, these microhabitats were sampled intensively during the second study period, in addition to accumulated soil in cavities from a fallen tree trunk and low light microhabitats from a residence interior.

During the second period of this study, a total of 1,523 soil samples were collected from different microhabitats (Table 1). The direct-observation and flotation techniques yielded a total of 64 sand flies, including 40 emerged adults, 16 larvae and 8 pupae. Of 58 emerged adults or immature forms that reached the adult form, 48 were identified as *L. longipalpis*, whereas 10 were not identified at the species level.

A total of 2,002 sand flies were collected with light traps during the second period of the study. Species in addition to those found in the soil samples were identified as follows: 9 *L. evandroi*, 2 *L. fischeri*, 82 *L. lenti*, 1,907 *L. longipalpis,* and 2 *L. peresi* (Table 2).

The yield of adult sand flies captured by HP light traps and the average temperature from the preceding week were associated at a moderated strength of association (Table 3). However, only the average temperature of the preceding week was found to be correlated significantly. All other climate variables in the preceding weeks were not significantly correlated with adult or immature sand flies yield (Table 4).

## 4. Discussion

Natural breeding places for phlebotomine sand flies, as the most likely places for the development of immature forms, may be represented by different microhabitats in different regions. In our study at Cavunge, a district with semiarid climate and Caatinga vegetation, positive soil samples were collected from a fallen trunk and between trees roots, as found in previous studies conducted at rain forests [5, 8]. Moreover, novel microhabitats, such as accumulated soil in rock cavities and from cracks around water tanks, were identified as positive for natural breeding sites.

Rock cavities in exposed rocks and cracks around a water tank, two of the most positive microhabitats, exhibit characteristics previously identified by Deane and M. P. Deane [9] and Newstead [7] as likely to favor the development of sand flies' immature forms. Rock cavities constitute microhabitats for larval development as they are poorly naturally illuminated. Additionally, water tanks are permanent filled with water, what leads to humidity and the presence of moist cracks in the soil.

As expected in a region affected by visceral leishmaniasis, most of the sand flies from the soil samples or captured by the light traps were identified as *L. longipalpis*. In studies conducted in rain forests [5, 8], several of the vectors of cutaneous leishmaniasis were abundant. However, *L. longipalpis* is often predominant in dry or urban locations in which visceral leishmaniasis occurs [13–20].

Analysis of the relationships between sand fly density and climate variables resulted in moderated strength association between adult sand flies captured and the average temperature from the preceding weeks. Such relationship was not observed by previous studies of associations between sand flies density and climate variables, which identified the absence of climatological influence in sand flies density [11, 12], or a trend for increasing soon after raining seasons [13, 14]. This novel possible relation contributes to the still uncertain framework of possible climate variables that interferes in the sand flies density in a dry climate region.

The analyses among climate data and sand flies density made by our study suffered limitations, because data gathering was not made *in loci*, but at approximately 40 km from the study site. Likewise, the absence of replication for the microhabitats sampled was an impediment to further statistical analyses that could lead to a comparison among adult and immature sand flies density. However, our study has identified several microhabitats as natural breeding sites and elucidated some possible relationships between vector density and climate variables that jointly should provide useful information from preferred periods and sites for application of insecticides.

## 5. Conclusions

The sand flies natural breeding places, as well as climate variables associated with vectors density, despite their potential to assist the biological control, are poorly understood so far. Our study points to several microhabitats that may serve as natural breeding places for phlebotomine sand flies in a semiarid region and also elucidate that temperature decreasing may be a predictor for the increasing of vector density in a dry climate region. Altogether, our results may furnish a tool for optimizing the control of the sand flies, and consequently, the anthropic and zoonotic cases of visceral leishmaniasis.

## Figures and Tables

**Table 1:** Positive microhabitats and sand flies yield.

Microhabitat	Collected samples	Positive samples	Sand flies
Chicken feces	87	1	1
Fallen tree trunk	55	5	14
House interior	105	1	1
Rock cavities	521	21	24
Tree roots	371	9	11
Water tank	384	12	13

Total	1,523	49	64

**Table 2:** Species of sand flies identified following sampling of adults.

Species	Inside houses		Adjacent to houses		Total
	M	F	M	F	
*L. evandroi*	2	1	4	2	9
*L. fischeri*	2	0	0	0	2
*L. lenti*	16	36	6	24	82
*L. longipalpis*	181	685	237	804	1,907
*L. peresi*	2	0	0	0	2

Total	203	722	247	830	2,002

**Table 3:** Multiple-regression analysis among climate variables and sand flies yield from sampling of adults.

Variable	R²	R² (adjusted)	*P *value
Average temperature			
1 week	43.6%	35.1%	0.016
2 weeks			0.134
3 weeks			0.964
Average rainfall (mm)			
1 week	10.0%	0.0%	0.216
2 weeks			0.664
3 weeks			0.747
Average rainfall (days)			
1 week	14.4%	1.5%	0.543
2 weeks			0.470
3 weeks			0.626
Average humidity			
1 week	16.4%	3.9%	0.500
2 weeks			0.652
3 weeks			0.910

**Table 4:** Multiple-regression analysis among climate variables and sand flies yield from soil sampling.

Variable	R²	R²(adjusted)	*P* value
Average temperature	9.5%	0.0%	
1 week			0.740
2 weeks			0.825
3 weeks			0.895
Average rainfall (mm)	1.4%	0.0%	
1 week			0.903
2 weeks			0.938
3 weeks			0.845
Average rainfall (days)	9.7%	0.0%	
1 week			0.579
2 weeks			0.253
3 weeks			0.640
Average humidity	13.5%	0.5%	
1 week			0.535
2 weeks			0.422
3 weeks			0.626
